# Single-cell sequencing and establishment of an 8-gene prognostic model for pancreatic cancer patients

**DOI:** 10.3389/fonc.2022.1000447

**Published:** 2022-09-28

**Authors:** Xiao Yu, Qiyao Zhang, Shuijun Zhang, Yuting He, Wenzhi Guo

**Affiliations:** ^1^ Department of Hepatobiliary and Pancreatic Surgery, The First Affiliated Hospital of Zhengzhou University, Zhengzhou, China; ^2^ Key Laboratory of Hepatobiliary and Pancreatic Surgery and Digestive Organ Transplantation of Henan Province, The First Affiliated Hospital of Zhengzhou University, Zhengzhou, China; ^3^ Open and Key Laboratory of Hepatobiliary & Pancreatic Surgery and Digestive Organ Transplantation at Henan Universities, The First Affiliated Hospital of Zhengzhou University, Zhengzhou, China; ^4^ Henan Key Laboratory of Digestive Organ Transplantation, The First Affiliated Hospital of Zhengzhou University, Zhengzhou, China

**Keywords:** single-cell sequencing, pancreatic cancer, chemokines, immune microenvironment, prognostic model

## Abstract

**Background:**

Single-cell sequencing (SCS) technologies enable analysis of gene structure and expression data at single-cell resolution. However, SCS analysis in pancreatic cancer remains largely unexplored.

**Methods:**

We downloaded pancreatic cancer SCS data from different databases and applied appropriate dimensionality reduction algorithms. We identified 10 cell types and subsequently screened differentially expressed marker genes of these 10 cell types using FindAllMarkers analysis. Also, we evaluated the tumor immune microenvironment based on ESTIMATE and MCP-counter. Statistical enrichment was evaluated using Gene Ontology and Kyoto Encyclopedia of Genes and Genomes pathway analysis. We used all candidate gene sets in KEGG database to perform gene set enrichment analysis. We used LASSO regression to reduce the number of genes in the pancreatic risk model by R package *glmnet*, followed by rtPCR to validate the expression of the signature genes in different pancreatic cancer cell lines.

**Results:**

We identified 15 cell subpopulations by dimension reduction and data clustering. We divided the 15 subpopulations into 10 distinct cell types based on marker gene expression. Then, we performed functional enrichment analysis for the 352 marker genes in pancreatic cancer cells. Based on RNA expression data and prognostic information from TCGA and GEO datasets, we identified 42 prognosis-related genes, including 5 protective genes and 37 high-risk genes, which we used to identified two molecular subtypes. C1 subtype was associated with a better prognosis, whereas C2 subtype was associated with a worse prognosis. Moreover, chemokine and chemokine receptor genes were differentially expressed between C1 and C2 subtypes. Functional and pathway enrichment uncovered functional differences between C1 and C2 subtype. We identified eight genes that could serve as potential biomarkers for prognosis prediction in pancreatic cancer patients. These genes were used to establish an 8-gene pancreatic cancer prognostic model.

**Conclusions:**

We established an 8-gene pancreatic cancer prognostic model. This model can meaningfully predict prognosis and treatment response in pancreatic cancer patients.

## Introduction

Pancreatic cancer is one of the most lethal malignancies and is associated with a high mortality rate (1–3). Pancreatic ductal adenocarcinoma (PDAC) is derived from the pancreatic ductal epithelium and accounts for about 90% of pancreatic cancer (4, 5). The lethality of pancreatic cancer is largely due to its difficulty to diagnose early and a lack of effective treatments. Despite improved surgical techniques and the use of neoadjuvant and adjuvant chemotherapies, the prognosis of pancreatic cancer remains stubbornly poor. Though immunotherapies have shown preliminary effectiveness in pancreatic cancer treatment, they are still at the preclinical stage (6). Therefore, it is urgent and imperative to explore the mechanisms of pancreatic cancer progression to identify new therapeutic modalities, especially immunotherapy.

The continuous development of sequencing technology has significantly improved RNA sequencing (RNA-seq) methods and techniques (7). However, bulk RNA-seq is the whole observation and detection of a population of cells. As the analysis represents the average expression of a population of cells, bulk RNA-seq fails to capture cellular heterogeneity (8, 9). The genetic information of cells with the same phenotype may differ significantly, and many rare cell populations are lost in the overall characterization. The emergence and advancement of single-cell sequencing (SCS) in the last decade have enabled precision genetic analysis of tumors (10, 11). SCS provides an abundance of molecular information, making it possible to characterize a variety of rare or previously unidentified cell populations within tumors (12, 13). SCS is a highly impactful tool that can facilitate the early diagnosis, tracking, and individualized treatment of cancers. SCS has been widely used to uncover molecular mechanisms and characterize rare subpopulations in many cancers, including lung cancer (14), breast cancer (15), gastric cancer (16), and colorectal cancer. In colorectal cancer, macrophages and dendritic cells were significantly associated with myeloid-targeting immunotherapies (17). SCS also revealed rare targetable genes that could be informative to develop lung cancer treatment (18, 19). However, SCS has been under-utilized in pancreatic cancer studies.

In the present study, we downloaded pancreatic cancer SCS data as well as corresponding clinical data from Gene Expression Omnibus (GEO) database, Cancer Genome Atlas (TCGA) expression data collection, and International Cancer Genome Consortium (ICGC). Based on these data, we identified 2 molecular subtypes of HCC with significant immunological differences. Then, we performed appropriate gene selection using dimensionality reduction algorithms. We examined molecular and immune signatures and constructed a polygenic risk score model. Our 8-gene model may serve as a promising biomarker to predict pancreatic cancer patient prognosis and immunotherapy efficacy.

## Methods

### Data acquisition and preprocessing

We downloaded the SCS data GSE156405 from GEO (http://www.ncbi.nlm.nih.gov/geo/) database. We retained scRNA-seq data from five patients with needle biopsy. We download pancreatic cancer gene expression spectrum data and clinical information from TCGA database (https://portal.gdc.cancer.gov/).

PACA-AU data was obtained from the ICGC database (https://dcc.icgc.org/). We obtained GSE21501, GSE28735, GSE57495, GSE62452, GSE71729, and GSE85916 datasets from the GEO database (https://www.ncbi.nlm.nih.gov/geo/). Subsequently, preserved pancreatic cancer sample data of six datasets (GEO) were combined and eliminated batch effects using removeBatchEffect (20).

### Dimensionality reduction

The scRNA-seq data were processed as described below:

1) Single-cell analysis was performed on five samples from GSE156405.
2) Set thresholds such that each gene was expressed in a minimum of three cells and each cell expressed a minimum of 250 genes.
3) The percentage of mitochondria and rRNA was calculated by PercentageFeatureSet function (21).
4) Cells were filtered such that each cell used in subsequent analysis expressed more than 500 and fewer than 6000 genes, had less than 30% mitochondrial gene expression, and a minimum unique molecular identifier (UMI) of 1000.
5) The FindVariableFeatures function was used to find hypervariable genes (21).

### Definition and analysis of cell subsets

The cells were clustered by FindNeighbors and FindClusters (21). We obtained immune cell markers associated with pancreatic cancer from previous studies, including stellate cell (*ADIRF*), fibroblasts (*COL1A1*, *COL1A2*, and *DCN*), T cells (*CD2*, *CD3D*, *CD3E*, and *CD3G*), B cells (*CD79A* and *CD79B*), neutrophils (*CSF3R*, *S100A8*, and *S100A9*), mast cells (*GATA2*, *TPSAB1*, and *TPSB2*), NK cells (*KLRF1*, *FGFBP2*, and *KLRC1*), macrophage (*CD163* and *CD68*), pDC (*LILRA4*) and pancreatic cancer cells (*KRT19* and *TM4SF1*) (22–24). FindAllMarkers was used to select marker genes of each subpopulation (logFC =0.5, Minpct=0.35). Prognostic genes were clustered by ConsensusClusterPlus in the RNA-seq cohort (25). The optimal number of clusters was determined according to the cumulative distribution function (CDF).

### DEGs identification and functional enrichment analysis

The *limma* package in R was used to identify differentially expressed genes (DEGs) associated with tumorigenesis by comparing gene expression levels between pancreatic cancer tissues and normal tissues. Statistical enrichment was evaluated using Gene Ontology (GO) and Kyoto Encyclopedia of Genes and Genomes (KEGG) pathway analysis. GO enrichment analyses included biological process (BP), molecular function (MF), and cellular component (CC).

### Cell score

CIBERSORT was used to calculate the immune cell fraction for each tumor subtype in the RNA-seq and GEO datasets. The MCP counter was used to evaluate the immune microenvironment, and the expression levels of immune cell marker genes were used to evaluate the degree of immune cell infiltration.

### LASSO regression and immune score

LASSO regression (developed by Tibshirani) can develop a refined model by constructing a penalty function (26, 27). It is a biased estimation with complex collinearity. In our study, we used LASSO regression to reduce the number of genes in the risk model by R package *glmnet* (28). Then, we performed a 10-fold cross-validation for model building. The ESTIMATE database was utilized to calculate immune cell score. CIBERSORT is a deconvolution algorithm that calculates the proportion of cells *via* processing of bulk RNA-seq data.

### Statistical analysis

We used SPSS 23.0 (SPSS, Inc., Chicago, IL) for statistical analysis. R package *Survival CoxPH* function was used to conduct a univariate Cox proportional risk regression model. Multivariate Cox regression analysis was performed on the prognosis-related genes identified by LASSO. The R package *timeROC* was used to perform receiver operating characteristic (ROC) analysis on the prognosis classification of the RiskScore. Differences were considered statistically significant at P < 0.05 (*P < 0.05; **P < 0.01; ***P < 0.001; ****P < 0.0001)

## Results

### Single-cell data for dimensionality reduction and clustering

**Figure 1:**
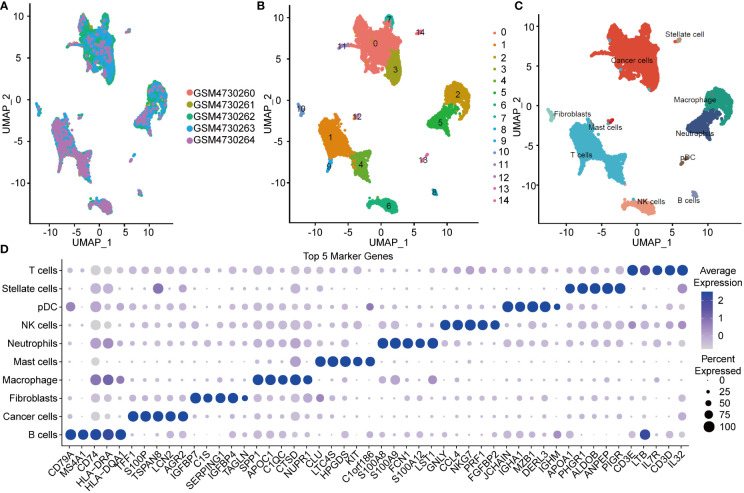
UMAP plot and maker genes of cell population. **(A)** UMAP plot of SCS in five samples. **(B)** UMAP of the 14 subgroups after clustering. **(C)** UMAP of subpopulation after cell annotation. **(D)** Top five marker genes in the cell population.

**Figure 2:**
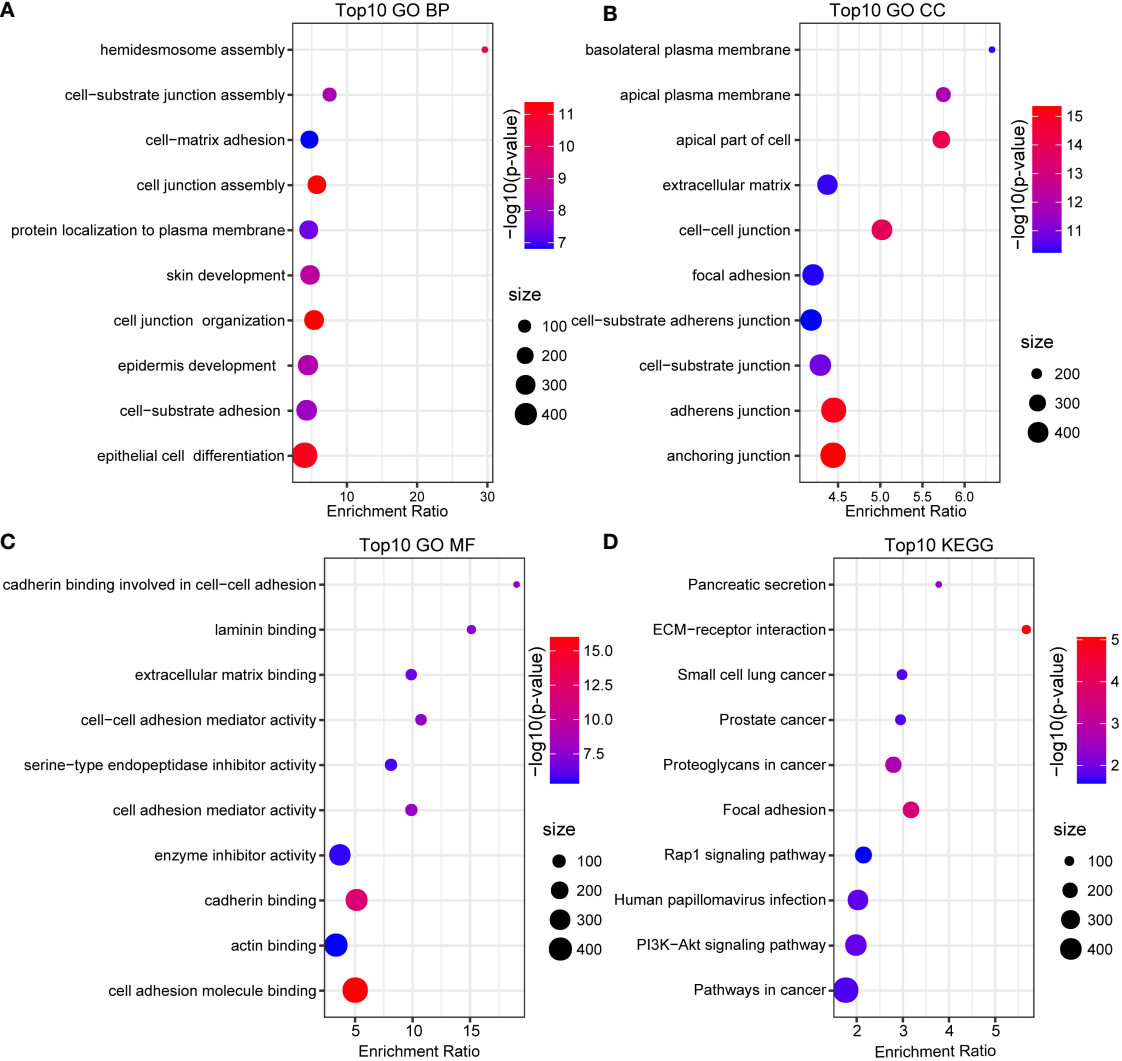
GO and KEGG annotation. **(A)** BP annotation of the maker genes in cancer cells. **(B)** CC annotation of the maker genes in cancer cells. **(C)** MF annotation of the maker genes in cancer cells. **(D)** KEGG annotation of the maker gene in cancer cells.

All data processing and analysis workflows were shown in **Figure S1**. We performed log-normalization to normalize data from five pancreatic cancer samples (**Figure 1A**). Through data integration, scaling, and PCA dimensionality reduction, we identified 15 distinct subpopulations (**Figure 1B**). The 15 subpopulations were re-grouped into 10 different cell types according immune cell marker gene expression (**Figure 1C**). We screened differentially expressed marker genes of the 10 cell types by FindAllMarkers analysis. The top five marker genes in each cell subpopulation were presented in **Figure 1D** **(positive=TRUE)**. Furthermore, we performed functional enrichment analysis using the 352 marker genes in pancreatic cancer cells. GO and KEGG pathway enrichment analyses were performed on the 352 DEGs using WebGestaltR tools (P<0.05) (29). According to GO functional annotation, 256 clusters were annotated significantly in the BP category (**Figure 2A**). A total of 161 GO terms were annotated in the CC category (**Figure 2B**). Lastly, 38 clusters with significant differences were annotated in the MF category (**Figure 2C**). Thirty-three DEGs were annotated in the KEGG pathway (**Figure 2D**). The results showed that intercellular junctions and extracellular matrix interactions were significantly enriched.

### Construction of pancreatic cancer molecular subtypes

**Figure 3:**
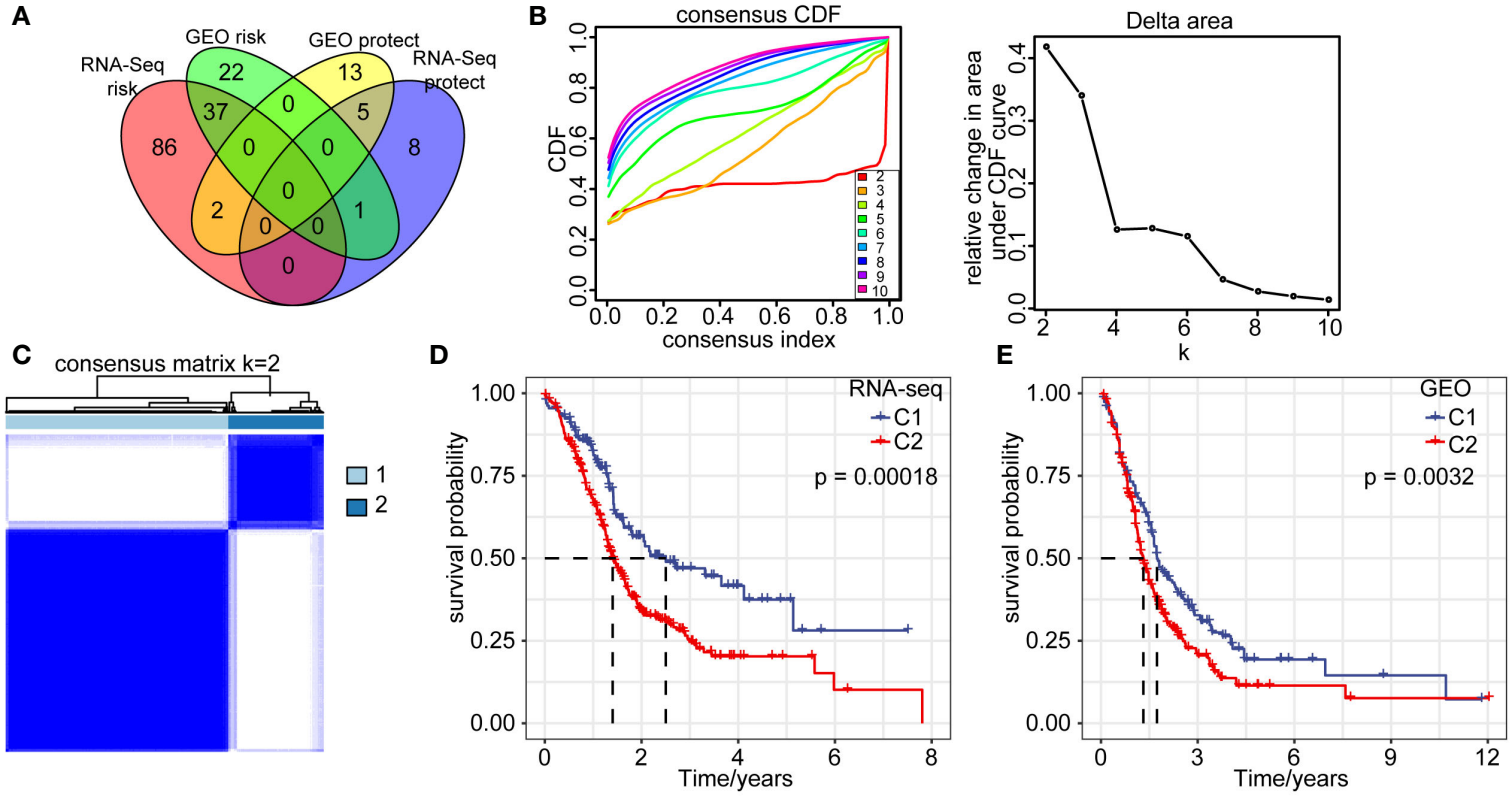
Molecular signatures of cancer cells. **(A)** Venn diagram overlap of 352 marker genes in cancer cells from two cohorts by single factor analysis. **(B)** CDF curve and CDF delta area of RNA-seq queue sample. **(C)** Heatmap of RNA-seq sample clustering (consensus k=2). **(D)** Overall survival curves of C1 and C2 subtype in RNA-seq dataset. **(E)** Overall survival curves of C1 and C2 subtype in GEO database.

The above analyses revealed cancer cells represented a large proportion of the cells sequenced from pancreatic cancer patients, as they were the main principal components. We further identified prognosis-related marker genes and constructed new molecular subtypes of pancreatic cancer based on them. We identified 5 protective genes and 37 high risk genes through univariate analysis in RNA-seq and GEO datasets (p < 0.05, HR > 1) (**Figure 3A**). Pancreatic cancer samples in RNA-seq were clustered by using 42 prognostic associated genes. CDF Delta area curve revealed the clustering result is relatively stable when the clustering number is two. Therefore, we defined 2 molecular subtypes (k=2) (**Figures 3B, C**). C1 subtype had a relatively better prognosis, whereas C2 subtype was associated with a worse prognosis. Similar results were also observed in the GEO cohort (**Figures 3D, E**), which further confirmed the prognostic significance of the molecular subtype classification.

### Cell type differences between C1 and C2 subtypes

**Figure 4:**
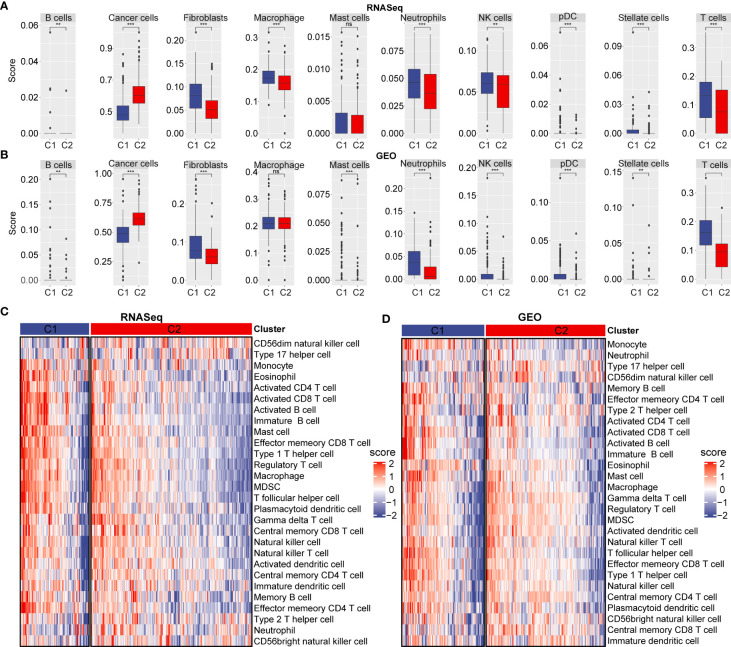
Immune cell scores and heat map of immune microenvironment score. **(A)** Cell score between C1 and C2 subtype in RNA-seq dataset. **(B)** Cell score between C1 and C2 subtype in GEO database. **(C)** Differences in heat map distribution of ssGSEA immune microenvironment score in subtypes. **(D)** Heatmap distribution differences of ssGSEA immune microenvironment score in GEO dataset in subtypes. **p < 0.01; ***p < 0.001; ns, no significant.

To assess cell differences between C1 and C2 subtypes, we probed our SCS data and by examining differentially expressed marker genes in different molecular subtypes. Using our RNA-seq data and the GEO database, we calculated the immune cell score of each subtype and compared the resulting score between the C1 and C2 subtypes. The results showed the cancer cell score of the C2 subtype was significantly increased compared with the cancer cell score in C1 subtype (**Figures 4A, B**). T and B cell scores in C1 subtype were significantly higher than in the C2 subtype.

### C1 and C2 subtype immune signatures

**Figure 5:**
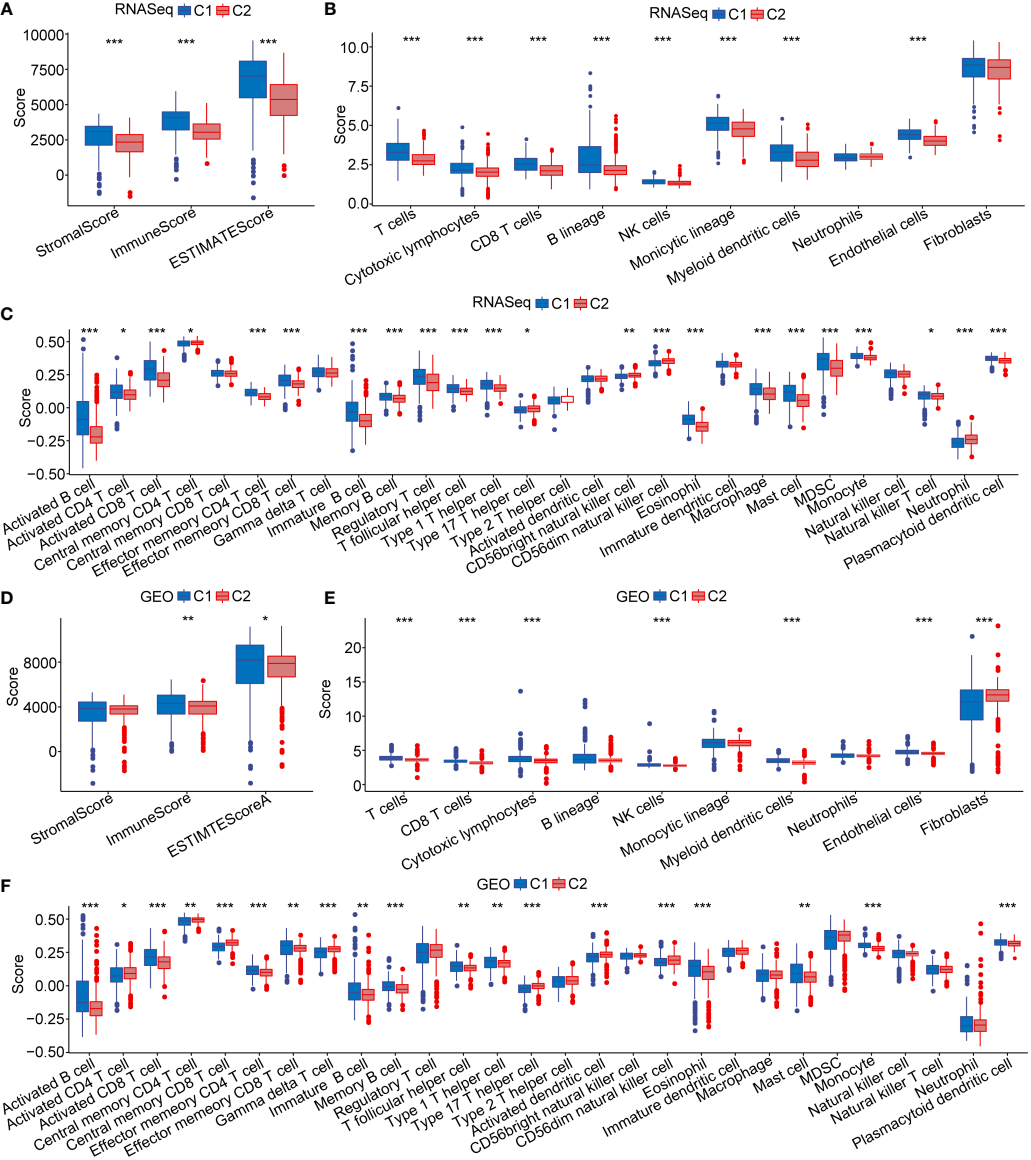
Immune microenvironment score. **(A–C)** The distribution of immune microenvironment score in RNA-seq dataset. **(D–F)** The distribution of immune microenvironment score in GEO database. *p < 0.05; **p < 0.01; ***p < 0.001.

To further explore immune microenvironment differences between the two subgroups, we evaluated the degree of immune cell infiltration in pancreatic· cancer cohorts using immune cell marker gene expression. The immune cell marker genes were derived from previous studies (30). The tumor immune microenvironment was also evaluated based on ESTIMATE and MCP-counter. The discrepancy of immune cells between the RNA-seq and GEO cohort was shown in **Figures 5A–F**. ESTIMATE, MCP-counter, and single sample gene set enrichment analysis (ssGSEA) revealed significant differences between most immune cells. The distribution of immune infiltration in RNA-seq was consistent with the GEO cohort. These results revealed the consistency of molecular characteristics and molecular subtype stability. We presented ssGSEA immune scores by heatmaps to illustrate differences in the immune microenvironment, (**Figures 4C, D**). Taken together, these findings demonstrated a significant difference in immune microenvironment score between C1 and C2 subtypes.

### Immune checkpoints and chemokines

The development and use of immune checkpoint targeting immunotherapies in the personalized treatment of pancreatic cancer has become increasingly common. Therefore, we evaluated the distribution of immune checkpoint gene expression in the RNA-seq and GEO datasets. We found 32 (69.57%) of the 46 checkpoint genes were aberrantly expressed in the RNA-seq dataset, and most of them were overexpressed in the C2 subtype, which has the worst prognosis (**Figures S2A**, **S2B**). In the GEO dataset, 28 (68.29%) of the 41 checkpoint genes were aberrantly expressed. Consistent with the RNA-seq dataset, most of them were overexpressed in the C2 subtype (**Figures S2C, S2D**).

Chemokines play essential roles in cancer progression and may facilitate immune cell migration into the tumor microenvironment, further affecting cancer progression and therapeutic response. Here, we analyzed the expression of chemokines in C1 and C2 subtype. In the RNA-seq data, 25 out of 41 chemokines were differentially expressed between the subtypes (**Figure S3A**), which suggest that the degree of immune cell infiltration in C1 and C2 subtypes may differ. In the GEO cohort, 22 out of 37 chemokines were differentially expressed between C1 and C2 subtype (**Figure S3B**). Such differences may contribute to rapid tumor progression and poor immunotherapy efficacy. Additionally, we evaluated chemokine receptor gene expression in the two subtypes. A total of 13 out of 18 chemokine receptor genes were differentially expressed in RNA-seq database (**Figure S3C**). In the GEO database, 11 of the 17 chemokine receptor genes were differentially expressed (**Figure S3D**). Chemokines and chemokine receptor relative gene expression maintained was consistent between C1 and C2 subtypes in both the RNA-seq and GEO datasets. Most were highly expressed in the C2 subtype, which is associated with a worst prognosis. These results also were illustrated as heat maps (**Figures S3E, S3F**).

### Functional and pathway enrichment analyses

To verify the functional differences between the C1 and C2 subtypes, we performed GO functional enrichment analysis of DEGs between the C1 and C2 subtypes in the RNA-seq dataset. Regarding GO function annotations of DEGs, 553 annotations with significant differences were observed in BP, including epithelial cell differentiation, angiogenesis, and regulation of signaling receptor activity (**Figure S4A**). A total of 124 GO CC terms were annotated (**Figure S4B**). The most significant difference between the C1 and C2 subtypes was plasma membrane protein complex. A total of 58 genes were significantly annotated in MF (**Figure S4C**). Additionally, KEGG pathway analysis was adapted for gene set enrichment analysis (GSEA) of C1 and C2 subtypes (p < 0.05, and FDR < 0.25) (**Figure S4D**) (31).

**Figure 6:**
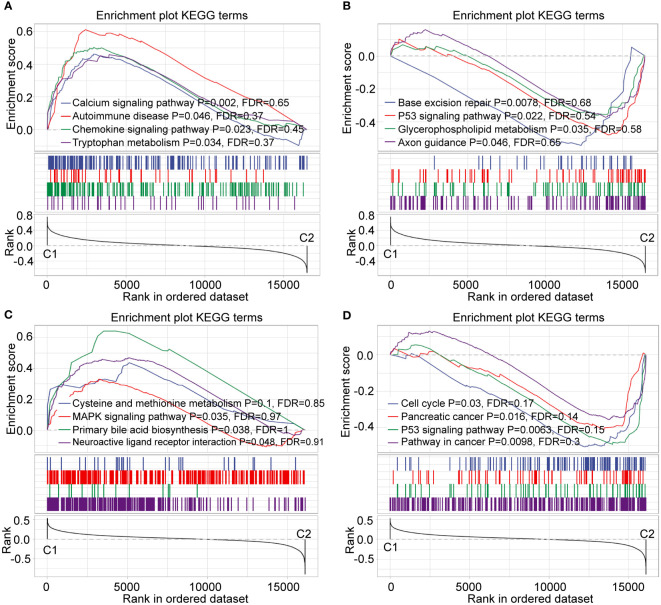
Enrichment analysis of GSEA pathway. **(A, B)** GSEA of C1 and C2 subtype in RNA-seq dataset. **(C, D)** GSEA of C1 and C2 subtype in the GEO dataset.

To continue exploring the C1 and C2 subtypes, we performed gene set enrichment analysis (GSEA) using all candidate gene sets in KEGG database. Several cancer-related pathways, such as base excision repair, P53 signaling pathway, and pancreatic cancer were significantly enriched in the C2 subtype according to the RNA-seq dataset (**Figures 6A, B**). Importantly, the enrichment results from the GEO dataset were consistent with the RNA-seq results (**Figures 6C, D**).

### Mutational signature and immune cell type classification

**Figure 7:**
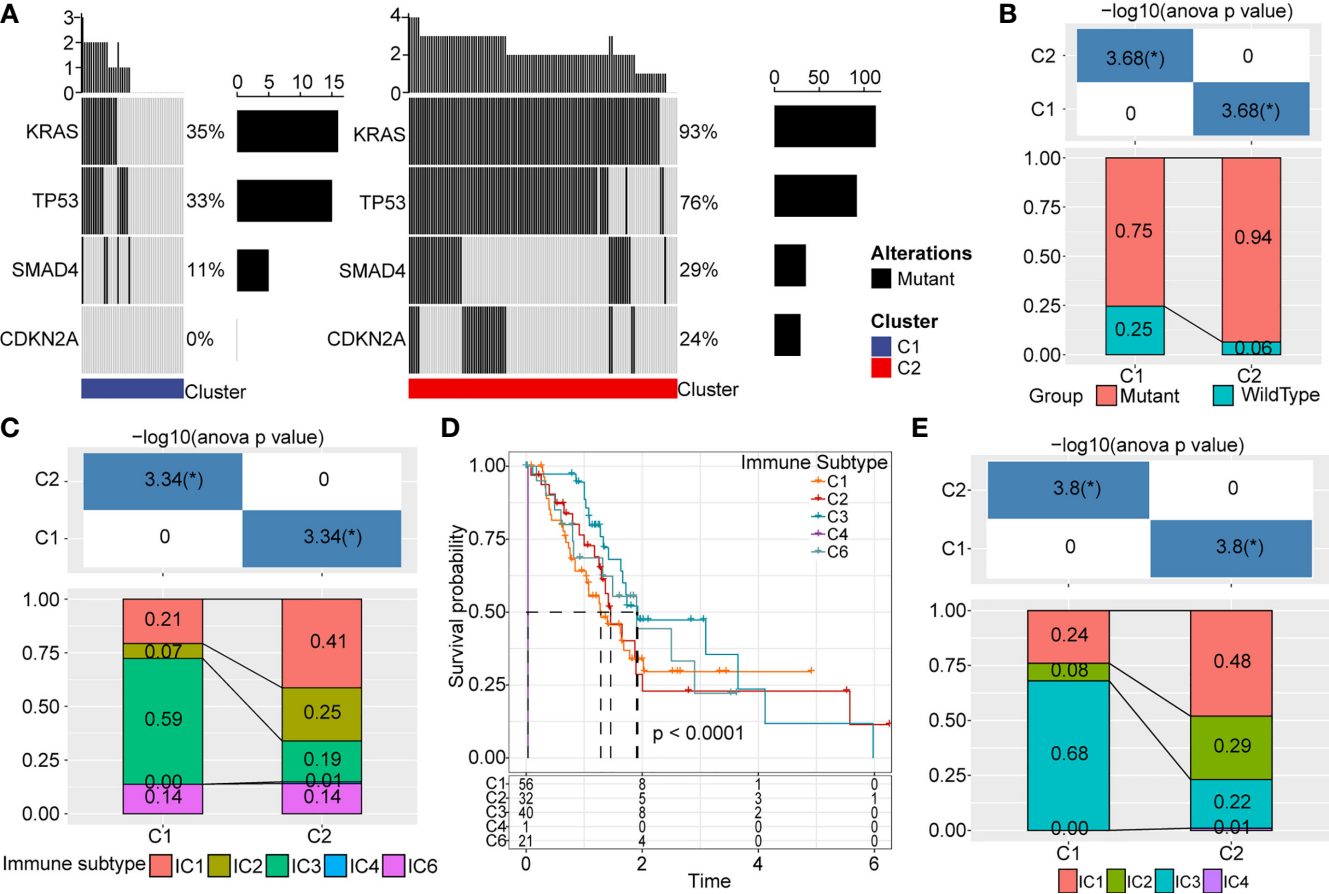
Gene mutations and distribution of immune subtypes. **(A)** Somatic mutations analysis of DEGs in two molecular subtypes. **(B)** Mutation frequency differences of KRAS among different subtypes in ICGC cohort. **(C)** Sankey between molecular types and immune subtypes. **(D)** Survival curve of existing immune subtypes. **(E)** Distribution of immune subtypes among different molecular types. *p < 0.05.

Also, we used the TCGA dataset to analyze gene mutations in the C1 and C2 subtypes. We found that subtype was markedly correlated with gene mutations in pancreatic cancer. We noted a higher percentage of KRAS, TP53, SMAD4, and CDKN2A mutations in the C2 subtype (**Figure 7A**). Similarly, data from ICGC-AU showed that the mutation frequency of KRAS was significantly lower in the C1 subtype than in the C2 subtype (**Figure 7B**).

Six infiltrating immune cell types have been identified in human cancer: IC1 (wound healing), IC2 (INF-R dominant), IC3 (inflammation), IC4 (lymphocyte depletion), IC5 (immunologically silenced), and IC6 (TGF-beta dominant). As expect, IC1, IC2, and IC6 are associated with poor prognosis. In TCGA, most pancreatic cancer patients belonged to IC1, IC2, and IC3 immune subtypes (**Figure 7C**), though the IC5 immune subtype is not included in TCGA data. Survival analysis revealed these immune subtypes were not significantly associated with overall survival in pancreatic cancer (**Figure 7D**). We also observed significant differences in the distribution of these immune subtypes in C1 and C2 subtypes (**Figure 7E**). IC3 was the major infiltrating immune cell type of the C1 subtype, whereas IC1 become the predominant immune cell type of the C2 subtype.

### Tumor Immune Dysfunction and Exclusion (TIDE) analysis

**Figure 8:**
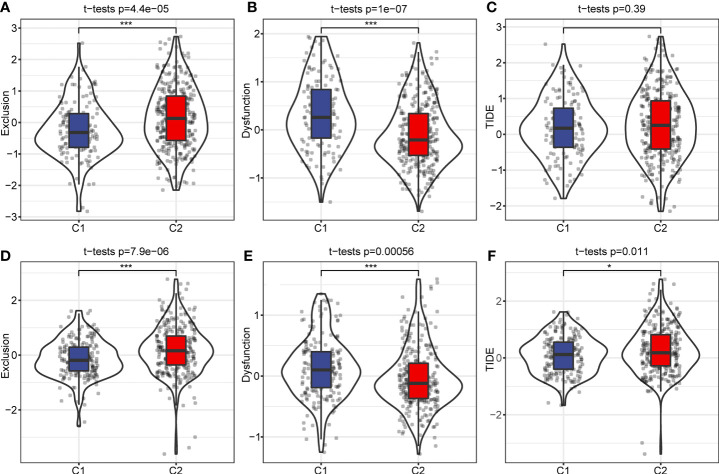
TIDE analysis of immune therapy. **(A)** Exclusion scores among different molecular subtypes in RNA-seq dataset. **(B)** C1 and C2 subtype dysfunction scores in the RNA-seq dataset. **(C)** TIDE score of different molecular subtypes in RNA-seq data. **(D)** Differences of Exclusion scores among different molecular subtypes of the GEO dataset. **(E)** C1 and C2 subtype Exclusion scores in the GEO dataset. **(F)** TIDE score of different molecular subtypes in the GEO dataset. *p < 0.05; ***p < 0.001.

We used the TIDE software (http://tide.dfci.harvard.edu/) to evaluate potential clinical effects of immunotherapy in the C1 and C2 subtypes. A higher TIDE prediction score indicates an increased chance of immune escape, which indicates immunotherapy may be less beneficial for these patients. In the RNA-seq dataset, exclusion scores were remarkably elevated in the C2 subtype and dysfunction scores were higher in the C1 subtype. However, TIDE scores between the C1 and C2 subtypes were not significantly different (**Figures 8A–C**). The exclusion and dysfunction scores in the GEO dataset were consistent with the RNA-seq dataset. Although, the TIDE score was increased in the C2 subtype compared to the C1 subtype (**Figures 8D–F**). This result was consistent with previous immune checkpoints and immune microenvironment analysis.

### Prognostic model

**Figure 9:**
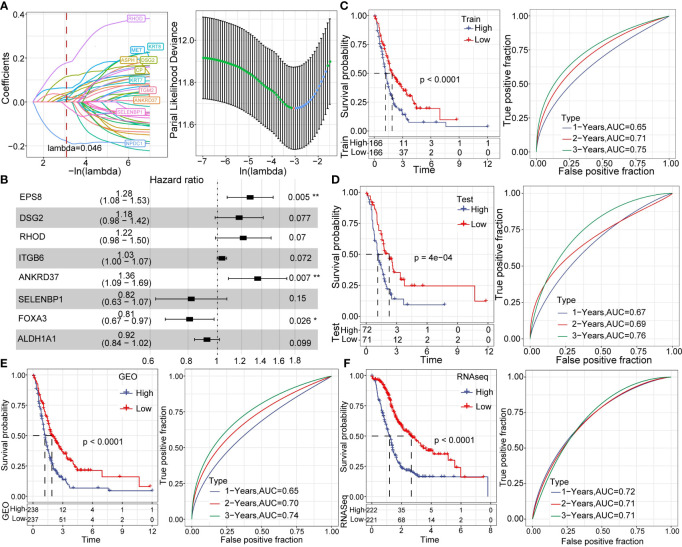
Establishment and analysis of prognostic models. **(A)** LASSO coefficient profiles of 42 prognostic genes in the GSE dataset. **(B)** Multivariate analysis of the risk model genes. **(C)** KM and ROC analysis of the risk model in the GEO dataset. **(D)** KM and ROC analysis of the risk model in the GEO validation dataset. **(E)** KM and ROC analysis of the risk model in the complete GEO dataset. **(F)** KM and ROC analysis of the risk model in complete the RNA-seq dataset. *p < 0.05; **p < 0.01.

Using the GEO dataset, we identified 42 genes that can predict cancer prognosis. We randomly sampled the GEO dataset with a sampling ratio of Train: test = 7:3. Then, we applied LASSO regression (lambda= 0.046) to obtain 11 prognosis-related genes (**Figure 9A**). To further reduce the number of genes, we used stepAIC, which reduced the number of genes to eight (*EPS8*, *DSG2*, *RHOD*, *ITGB6*, *ANKRD37*, *SELENBP1*, *FOXA3*, *ALDH1A1*) and determined the corresponding risk coefficients (**Figure 9B**) (32). Finally, we arrived at an 8-gene prognostic model for pancreatic cancer patients. We calculated the risk score of each sample in both the GEO training dataset and validation dataset and divided the samples into high and low risk groups using the median as cutoff. A higher score predicted poorer prognosis in pancreatic cancer patients. Kaplan Meier (KM) curves and ROC curves were displayed in **Figures 9C, D**. The corresponding AUC in 1-year, 2-year, and 3-year were 0.65, 0.71, and 0.75, respectively. Moreover, we validated our model in GEO validation, TCGA, and ICGC datasets (**Figures 9E, F**).

## Discussion

SCS technologies have been increasingly used in disease research over the past decade (33–35). SCS can reveal gene structure and expression status at single-cell resolution, which facilitated the exploration of biological processes and disease mechanisms with unprecedented precision (36, 37). In our study, we classified and defined two pancreatic cancer subtypes by analyzing SCS data from multiple public databases. We observed sequenced cells form 10 cell subgroups, including stellate cells, fibroblasts, T cells, B cells, neutrophils, mast cells, NK cells, macrophages, pDCs, and pancreatic cancer cells. These subgroups can be identified by expression of specific marker genes. Researchers have found immune cells of the various lymphoid and myeloid lineages by SCS (38). These immune cell subsets facilitated the formation of an immunosuppressive microenvironment in pancreatic cancer. We further explored the molecular and immune signatures of different cell subsets. Furthermore, we used TIDE software to evaluate potential clinical immunotherapy efficacy in the C1 and C2 subtypes. C2 had a higher TIDE score, which suggests patients with this subtype would benefit less from immunotherapy. Nevertheless, we need to note that the mechanism(s) of immune cell exclusion and dysfunction may well be different in different tumor types. Therefore, this conclusion still needs to be verified by more precise molecular and animal experiments.

Pancreatic cancer has an extremely poor prognosis, which is largely attributed to its difficulty to diagnose early (39–41). Given this limited therapeutic window, it is of great interest to establish gene models that can effectively predict pancreatic cancer prognosis. Hosein et al. (38) observed macrophage heterogeneity in pancreatic cancer at different stages using single-cell RNA-seq. Specifically, they revealed macrophages express different genes at different times and play different functional roles. Studies have yet to relate marker genes with cell subgroup in pancreatic cancer.

We identified 10 different cell types using data clustering and dimension reduction. Then, we performed functional enrichment analysis for 352 marker genes in pancreatic cancer cells. Notably, we found some marker genes were markedly correlated with pancreatic cancer progression. These finding indicate the prognostic risk model may provide clinical treatment guidance. Specifically, we found *EPS8*, *DSG2*, *RHOD*, *ITGB6*, *ANKRD37*, *SELENBP1*, *FOXA3*, and *ALDH1A1* could be potential biomarkers to accurately predict the prognosis of pancreatic cancer patients. Therefore, we established a prognostic model based on these eight genes. The prognostic risk model was a worthy prognostic indicator, as evidenced by its ability to accurately indicate prognosis in three external datasets (GEO validation, TCGA, and ICGC datasets). Similar with our findings, serval studies have demonstrated *EPS8*, *DSG2*, *ITGB6* were aberrantly expressed in pancreatic cancer, breast cancer, pituitary tumor, and gastric cancer (42, 43). Hütz et al. (43) found silenced *DSG2* facilitated pancreatic cancer cell migration and invasion. *EPS8* increased polyubiquitination by downregulating ALDH7A1 protein expression in pancreatic cancer. Zhuang et al. (44) observed that *ITGB6* was significantly upregulated and closely associated with overall survival in pancreatic cancer. These findings have important significance for prognosis prediction and subsequent treatment of pancreatic cancer patients. The risk model could predict the patient prognosis and may inform the use of individualized therapies in pancreatic cancer patients.

## Conclusion

Based on pancreatic cancer SCS data from the GEO, TCGA and ICGC databases, we identified a new molecular subtype of HCC with distinct molecular and immune signatures. Meanwhile, we established a risk model based on 8 prognosis-related genes, which has stable and effective prognosis prediction performance.

## Data availability statement

We downloaded the SCS data GSE156405 from GEO (http://www.ncbi.nlm.nih.gov/geo/) database. We retained scRNA data from five patients with needle biopsy. We download pancreatic cancer gene expression spectrum data and clinical information from TCGA database (https://portal.gdc.cancer.gov/). PACA-AU data was obtained from the ICGC database (https://dcc.icgc.org/). TCGA and ICGC datasets were combined to RNA-seq. Then, batch effects were eliminated using removeBatchEffect. We obtained GSE21501, GSE28735, GSE57495, GSE62452, GSE71729, and GSE85916 datasets from the GEO database (https://www.ncbi.nlm.nih.gov/geo/).

## Author contributions

WG, SZ, and YH designed and guided the study. XY and QZ analyzed the data and wrote the manuscript. XY drew the figures. All authors read and approved the final manuscript.

## Funding

This work was supported by Leading Talents of Zhongyuan Science and Technology Innovation (214200510027), Henan Provincial Medical Science and Technology Research Plan (SBGJ2018002 and SBGJ202102117), Henan Medical Science and Technology Joint Building Program (LHGJ20210324), Outstanding Foreign Scientist Studio in Henan Province (GZS2020004), and the Gandan Xiangzhao Research Fund (GDXZ2022002).

## Conflict of interest

The authors declare that the research was conducted in the absence of any commercial or financial relationships that could be construed as a potential conflict of interest.

## Publisher’s note

All claims expressed in this article are solely those of the authors and do not necessarily represent those of their affiliated organizations, or those of the publisher, the editors and the reviewers. Any product that may be evaluated in this article, or claim that may be made by its manufacturer, is not guaranteed or endorsed by the publisher.
