# Supplementary figures and images for: Single-cell sequencing and establishment of an 8-gene prognostic model for pancreatic cancer patients
Source: Front Oncol. 2022 Sep 28;12:1000447. doi: 10.3389/fonc.2022.1000447 (PMC9552769; doi:10.3389/fonc.2022.1000447)

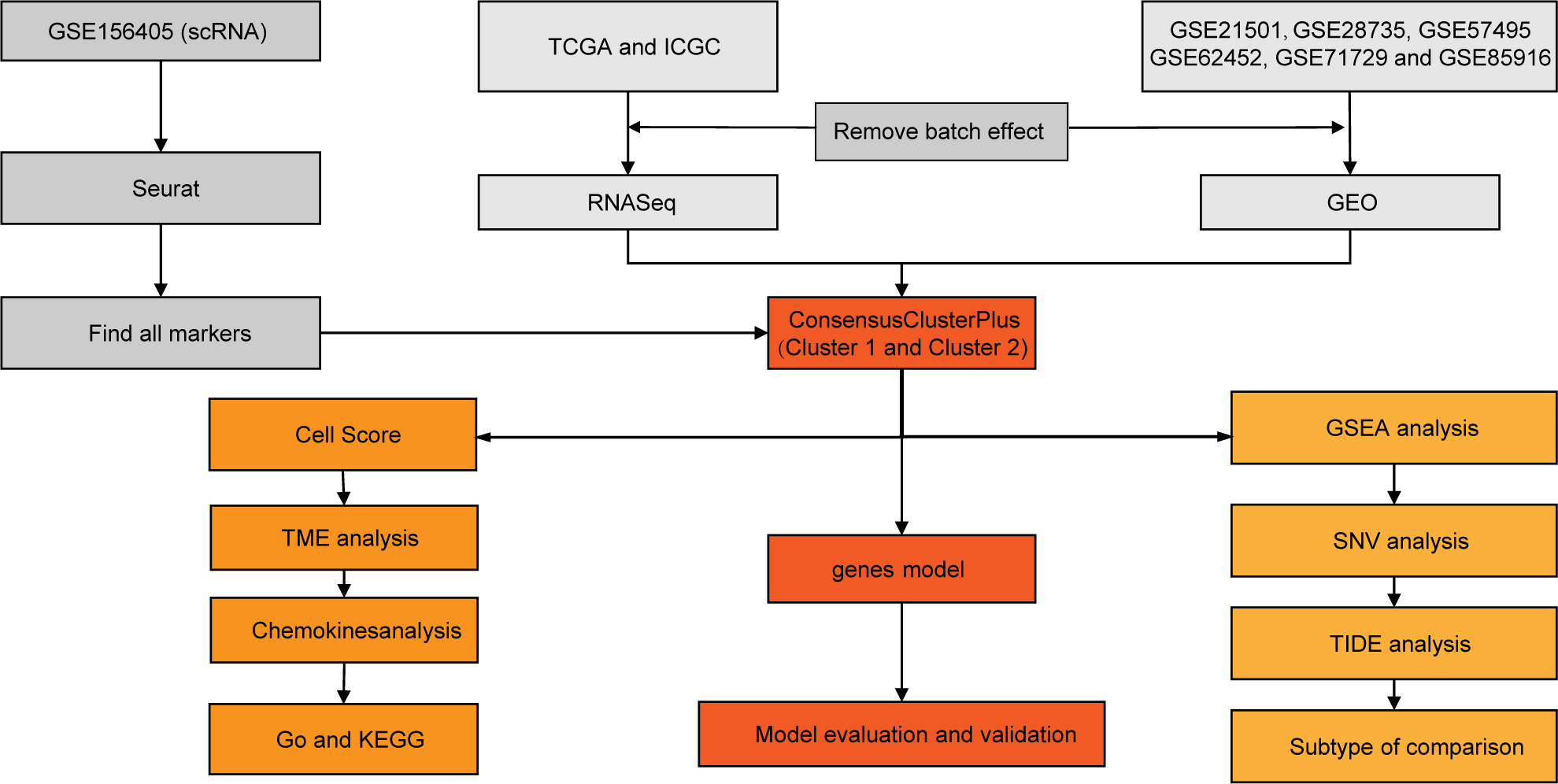

Supplement: Supplementary file 1 [file Image_1.tif]

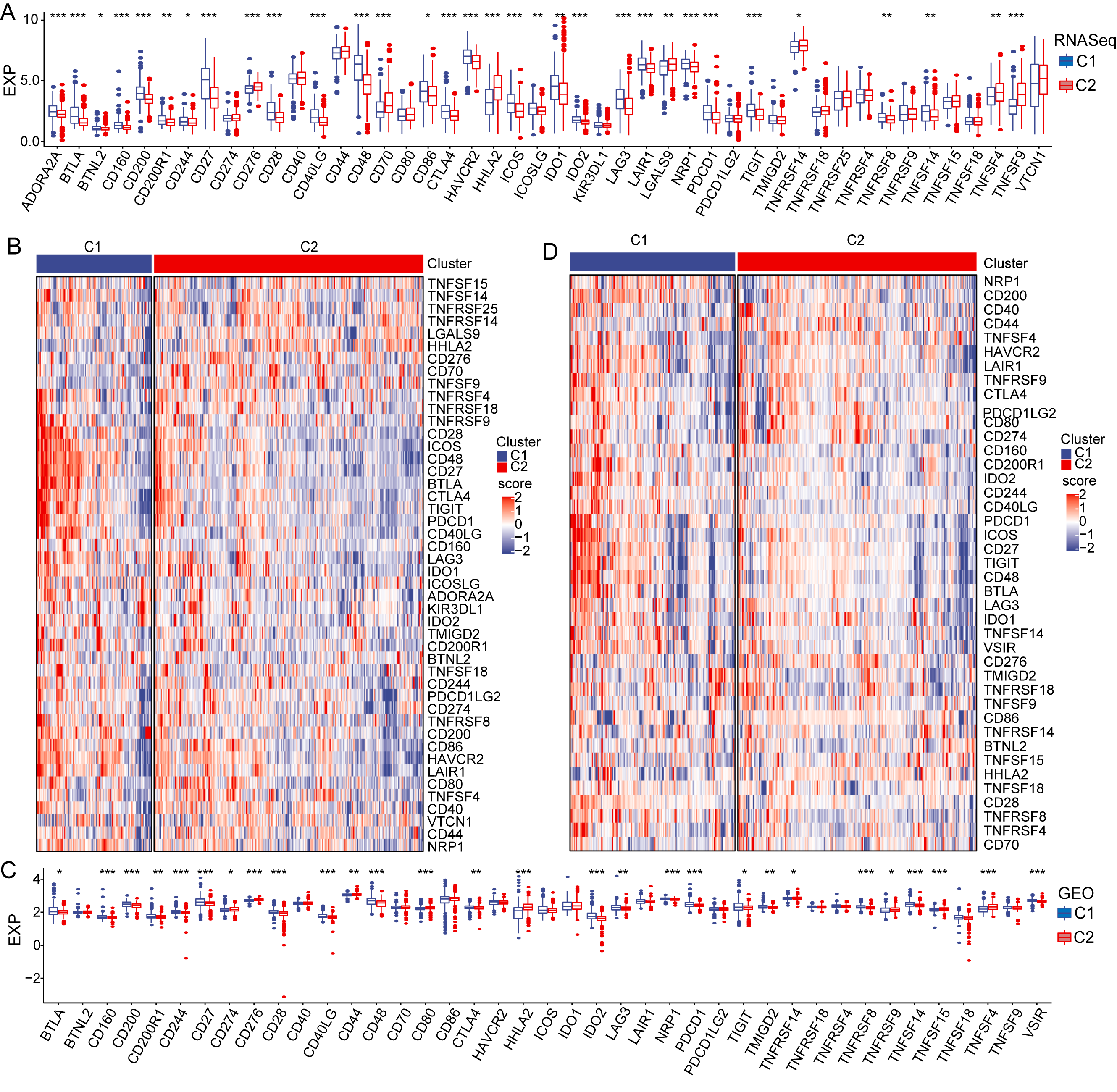

Supplement: Supplementary file 2 [file Image_2.tif]

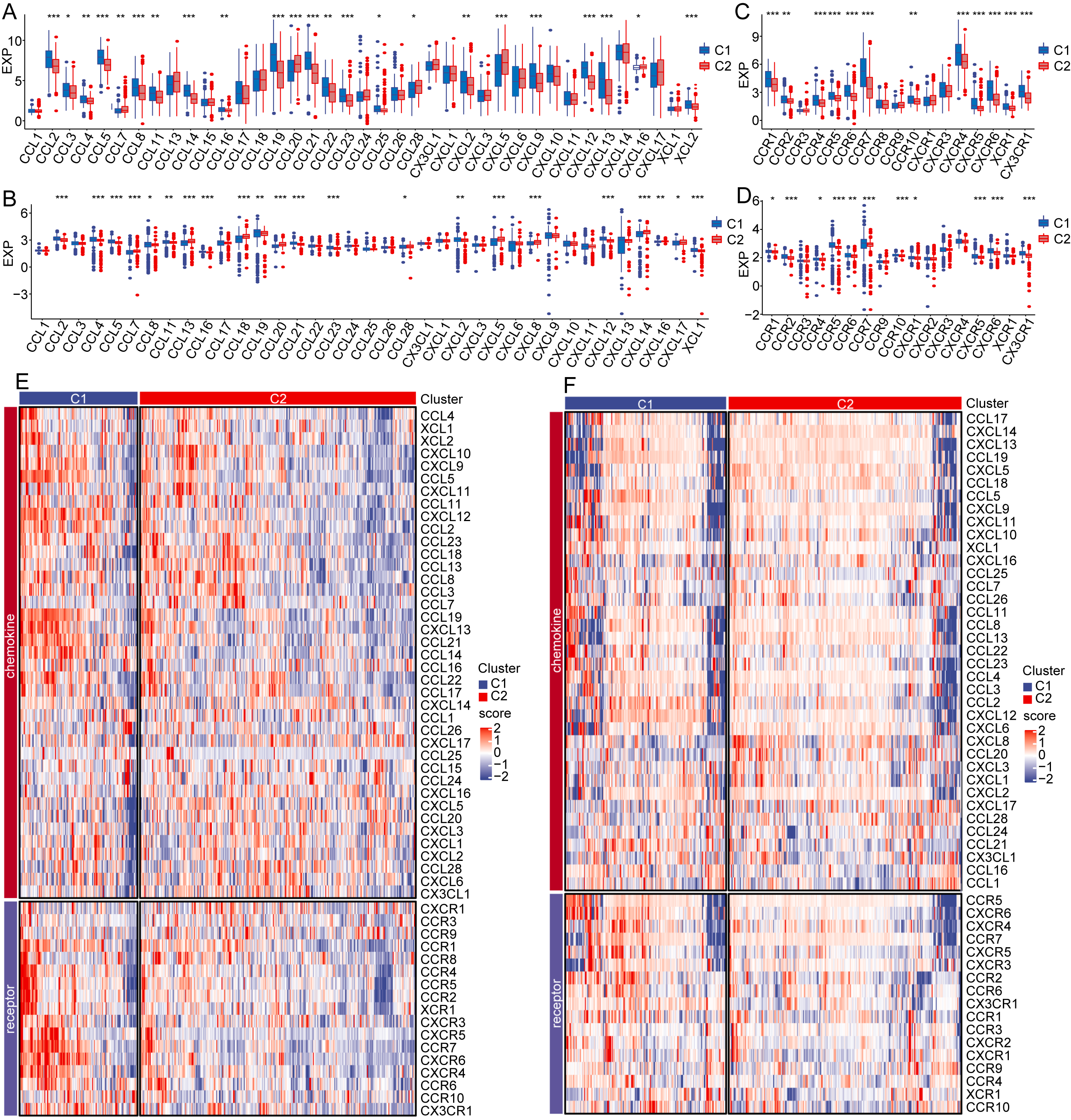

Supplement: Supplementary file 3 [file Image_3.tif]

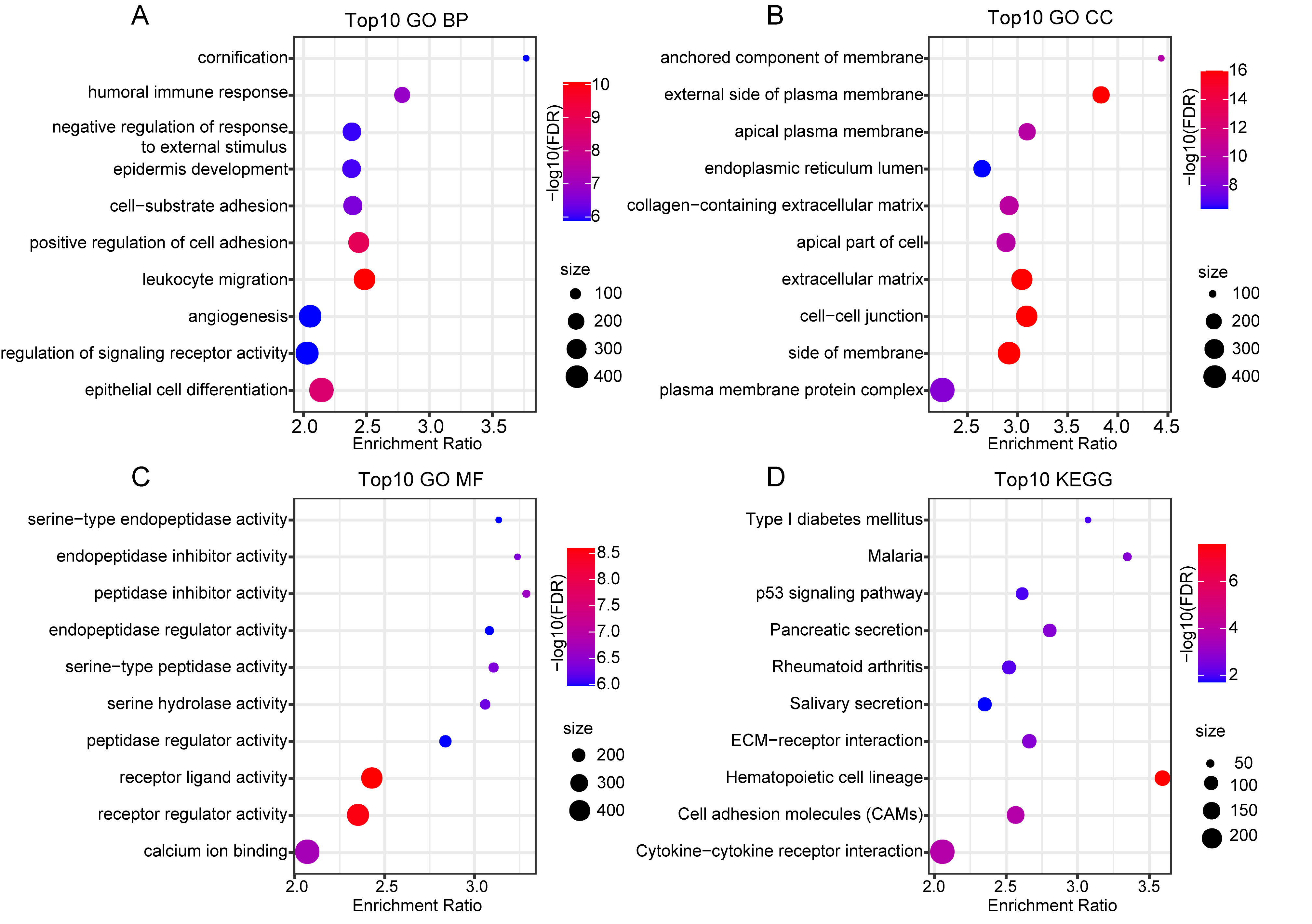

Supplement: Supplementary file 4 [file Image_4.tif]
