# Supplementary material for: Single-cell sequencing and establishment of an 8-gene prognostic model for pancreatic cancer patients
Source: Front Oncol. 2022 Sep 28;12:1000447. doi: 10.3389/fonc.2022.1000447 (PMC9552769; doi:10.3389/fonc.2022.1000447)
Supplement: Supplementary file 5 [file Table_1.docx]

**Fig S1. Flowchart of the study**

**Fig S2. Distribution of immune checkpoints. A.** The expression distribution of immune checkpoints of C1 and C2 subtype in the RNA-seq dataset. **B.** Heat map of distribution of immune checkpoints of C1 and C2 subtype in the RNA-seq dataset. **C.** The expression distribution of immune checkpoints of C1 and C2 subtype in the GEO dataset. **D.** Heatmap of distribution of immune checkpoints of C1 and C2 subtype in the GEO dataset.

**Fig. S3. Expression distribution of chemokine and** **chemokine receptors.** **A.** The expression distribution of chemokine in RNA-seq cohort. **B.** The expression distribution of chemokine in the GEO dataset. **C.** The expression distribution of chemokine receptor in the RNA-seq cohort. **D.** The expression distribution of chemokine receptor in the GEO dataset. **E.** Heatmap of the distribution of chemokine and chemokine receptors of C1 and C2 subtypes in the RNA-seq dataset. **F.** Heatmap of the distribution of chemokine and chemokine receptors of C1 and C2 subtype in the GEO dataset.

**Fig. S4.** **GO and KEGG annotation of DEGs**. **A.** BP annotation of DEGs between C1 and C2 subtypes in the RNA-seq dataset. **B.** CC annotation of DEGs between C1 and C2 subtypes in the RNA-seq dataset. **C.** MF annotation of DEGs between C1 and C2 subtypes in the RNA-seq dataset. **D.** KEGG annotation of DEGs between C1 and C2 subtypes in RNA-seq data.
